# 4051 Assessing outcomes of Miami CTSI’s Mentored Career Development KL2 Program: Using bibliometric and network visualization approaches to complement traditional outcome metrics

**DOI:** 10.1017/cts.2020.230

**Published:** 2020-07-29

**Authors:** Rosalina Das, Jessica Diaz, Patricia Avissar, Tatjana Rundek, Gwendolyn B. Scott, Alessia Fornoni, Jonelle E. Wright, Sheela Dominguez, Barry S. Issenberg, Ralph L. Sacco

**Affiliations:** 1 University of Miami Clinical and Translational Science Institute; 2 University of Miami

## Abstract

OBJECTIVES/GOALS: The goal of this project was to assess the scientific impact of Miami CTSI’s Mentored Career Development (KL2) Program using bibliometric tools and network visualization in addition to the traditional metrics used to provide a comprehensive evaluation. METHODS/STUDY POPULATION: Scholarly productivity of KL2 scholars were tracked using REDCap. For bibliometric data analysis and visualization, publications were queried using iCite (NIH Office of Portfolio Analysis) and Web of Science database. A total of 173 publications produced by eight KL2 scholars from 2013-2018 were analyzed and categorized into pre-award, during award, and post-award periods. iCite was used to assess scientific influence and translation. Scientific networks and collaboration were visualized using VOSviewer (Centre for Science and Technology Studies, Leiden University). CTSA Common Metrics were tracked using the Results Based Accountability framework. RESULTS/ANTICIPATED RESULTS: Albeit of modest size, the Miami CTSI’s KL2 Program had significant scientific productivity and impact in its first five years. Our KL2 scholars’ publications were cited twice as frequently as other papers in their fields. Further, 48% of publications post KL2 award were above the NIH 50th percentile and had higher citation impact compared to the average NIH-funded paper; 11% were in the top 10% NIH citation ranking. In contrast, only 20% of the publications pre-KL2 award were above the NIH 50th percentile. The program also promoted research collaboration; network visualizations indicate larger co-authorship and organization networks of KL2 scholars post-award. DISCUSSION/SIGNIFICANCE OF IMPACT: Bibliometric and data visualization approaches helped us better identify trends and gauge effectiveness of the KL2 program. These findings provided useful insight into the scientific influence and impact of our scholars’ work.

